# *ESR1* and *ESR2* gene polymorphisms are associated with human reproduction outcomes in Brazilian women

**DOI:** 10.1186/s13048-014-0114-2

**Published:** 2014-12-20

**Authors:** Clarissa Santiago de Mattos, Camila Martins Trevisan, Carla Peluso, Fernando Adami, Emerson Barchi Cordts, Denise Maria Christofolini, Caio Parente Barbosa, Bianca Bianco

**Affiliations:** Human Reproduction and Genetics Center – Division of Sexual and Reproductive Healthcare and Population Genetics - Faculdade de Medicina do ABC, Av. Príncipe de Gales, 821, Santo André, SP 09060-650 Brazil; Division of Epidemiology and Biostatistics, Department of Collective Health - Faculdade de Medicina do ABC, Santo André, SP Brazil

**Keywords:** Estrogen receptor gene, Polymorphism, *In vitro* fertilization, Controlled ovarian hyperstimulation

## Abstract

**Background:**

Important candidate genes involved in the ovarian response to exogenous FSH are the estrogen receptor genes (ESRs), since the effects of estrogens on follicle growth, maturation and oocyte release. It is known that some markers of ovarian stimulation can help to personalize the treatment, adjusting the dose of exogenous rFSH, thus preventing excessive wear of the patient. Inspired on this information we aimed to analyze four different polymorphisms in the estrogen receptor genes *ESR1*: rs2234693/T-397C (*PvuII)* and rs9340799/A-351G (*Xbal)* and *ESR2*: rs4986938/G1082A *(RsaI*) and rs1256049/A + 1730G (AluI), and their association with assisted reproduction outcomes in Brazilian women that underwent *in vitro* fertilization (IVF).

**Methods:**

A cross-sectional study was performed involving 136 infertile women less than 39 years of age with normal ovarian reserve. Patients were divided according to the same COH protocol for statistical analysis. The Taqman assay was used for PvuII and XbaI of ESR1, and RsaI and AluI of ESR2 genotyping. Serum estradiol and FSH were measured by Elisa assay.

**Results:**

The PvuII (ESR1) TT and RsaI (ESR2) GG genotypes were associated with a longer induction period and higher doses of medication (p < 0.03). The XbaI (ESR1) AA genotype was associated with better COH results, including a larger number of follicles, mature oocytes, embryos, and good quality embryos (p < 0.05). The AluI GG genotype showed an association with the Ovarian Hyperstimulation Syndrome (OHSS) (p = 0.03). According to the haplotype analysis of ER1 (PvuII/XbaI), we demonstrated that the CA combination increases by 0.68 the number of good quality embryos while the TG decreases it by 0.71 (p = 0.04).

**Conclusion:**

ER polymorphisms have an association with the assisted reproduction outcomes in Brazilian women.

## Introduction

Infertility is a health problem that affects 15–20% of couples at a reproductive age [1], with several medical, emotional, and social implications. The use of *in vitro* fertilization (IVF) has improved the treatment prospects for infertility by diverse causes. In developed countries, it is estimated that 2-3% of all births are results of IVF procedures [2].

IVF is a multistep process that still has limited successful rates regardless of constant improvement in laboratory performance, as it also depends on interindividual variability factors such as ovarian response after controlled ovarian hyperstimulation (COH). Both quantitative and qualitative factors in oocyte production have a high influence on the IVF outcome, since increasing the number of mature oocytes improves the selection of viable embryos for transfer [3].

Although low response rates can lead to cycle cancellation, high responses are troublesome as they can trigger a serious medical iatrogenic condition – the ovarian hyperstimulation syndrome (OHSS). This unpredictable individual variability in the ovarian response to COH represents one of the most challenging issues of IVF treatment safety. Previously published data indicates that age, Follicle Stimulating Hormone (FSH), Estradiol, Inhibin B, Anti-Müllerian hormone (AMH) serum levels, and antral follicle count (AFC) are clinically relevant predictors of oocyte yield [4–8], but none of this markers have significant predictive value when considered alone [6,8].

Besides these parameters, a complementary strategy involves studying the pharmacogenetics of the COH response. Candidate genes involved in the reproductive system present single-nucleotide polymorphisms (SNPs) that can lead to amino acid change in the protein and/or affect gene expression, and seem to be an important factor in changing the cellular and tissue effect of gonadotropins [9].

Important candidate genes involved in the ovarian response to exogenous FSH are the estrogen receptor genes (ESRs), since the effects of estrogens on follicle growth, maturation, and oocyte release have been well reported [10]. Estrogens extend the action of FSH on granulosa cells by promoting their proliferation and increasing their expression of FSH receptors [11]. In addition, they also play a regulatory role in endometrial cell growth and differentiation in the reproductive cycles [12], and consequently, exert a crucial effect on endometrial preparation for implantation [13]. Blastocysts and two-cell embryos express estrogen receptors (ERs) mRNA, which supports the possibility that estrogens also play a role in early embryonic development [14].

Two subtypes of ERs have been identified in humans, ERα and ERβ, encoded by *ESR1* (6q25.1) and *ESR2* (14q23.2) genes, respectively. In folliculogenesis, *ERα* are expressed predominantly in the theca layer and mediates estrogen proliferative actions. *ERβ*, expressed in granulose cells of growing follicles in different stages of development, promotes the differentiation and antiproliferative effects required for reaching the antral stage [15].

Prior findings suggested that genetic variability of ESRs may be important for advances in the diagnosis and treatment of infertility. Associations with the ovarian response in COH/IVF have been studied the most, regarding two SNPs: rs2234693/T-397C (defined by the cleavage site of restriction enzyme *PvuII*) and rs9340799/A-351G (defined by the cleavage site of restriction enzyme *XbaI*) in *ESR1,* and two other identified polymorphisms in *ESR2*: rs4986938/G1082A (defined by the cleavage site of restriction enzyme *RsaI*) and rs1256049/A + 1730G (defined by the cleavage site of restriction enzyme *AluI*) [1].

Inspired by these findings, the purpose of the present study was to analyze, for the first time in infertile Brazilian women, the association of *ESR1* (*PvuII* and *XbaI*) and *ESR2* (*AluI* and *RsaI*) polymorphisms with the controlled ovarian hyperstimulation response, as well as human reproductive outcomes in a normal ovarian reserve group with a fixed dose of recombinant FSH protocol.

## Material and methods

### Patients

A prospective cross-sectional study was performed on 380 women who underwent the first cycle of high-complexity IVF between September 2011 and September 2013 at the Human Reproduction and Genetics Center of the *Faculdade de Medicina do ABC, Santo André, São Paulo, Brazil.*

One hundred and thirty-six out of 380 patients met the inclusion criteria, which were age ≤38 years old, FSH ≤10mIU/mL, AMH ≥0.5 ng/mL, normal ovulatory cycles (25–35 interval days), and both ovaries present with no morphological abnormalities. We excluded patients with a history of inadequate ovulatory response, prior ovarian surgery, radio/chemotherapy, moderate/severe endometriosis (grades III and IV), and endocrine diseases such as hyperprolactinemia (PROL ≥25 ng/mL), thyroid dysfunction (TSH ≥4.5mIU/mL), obesity (body mass index ≥30), and polycystic ovary syndrome.

The indications for IVF were tubal factor infertility (33%), male factor infertility (54%), and unexplained infertility (13%). The investigation as to the cause of infertility included a complete medical history and physical examination, hormonal and biochemical profile, semen analysis of the partner, investigation of immunological and genetic abnormalities, hysterosalpingography, and hysteroscopy. All patients were investigated for endometriosis with laparoscopy performed if there were symptoms or abnormalities on the physical examination, CA 125 or imaging tests (ultrasound or/and pelvic magnetic resonance). In all women less than 36 years of age with no other infertility causes, laparoscopy was routinely performed. If the cause of infertility could not be diagnosed in spite of this evaluation, the case was considered unexplained. Anatomic tubal abnormalities were diagnosed with hysterosalpingography and/or laparoscopy, and male factor infertility was classified according to the criteria of the World Health Organization [16].

Written informed consent, approved by the local Ethics in Research Committee, was obtained from all patients participating in the present study.

### Ovarian stimulation protocol and IVF outcome

COH was conducted according to the GnRh (Gonadotropin Releasing Hormone) antagonist protocol with a fixed dose of recombinant FSH (rFSH) per day. The dose of rFSH was chosen between 100 or 200 IU according to age (100 IU below 37 years of age and 200 IU for patients 37 years of age or more), antral follicle count and/or prior inductions. All patients started with an injection of rFSH on day 2 of menstruation, continuing between 9 and 11 days when the two biggest follicles reached 18 mm in diameter. The GnRh agonist was initiated on the 6^th^ day or when at least one follicle had reached 14 mm. Cycles were canceled when follicles did not show any satisfactory response until the 8^th^day of rFSH. Final follicle maturation was achieved using 250 μg of hCG followed by oocyte retrieval 36 hours later.

The number of mature oocytes was calculated for both IVF and *in vitro* fertilization with sperm injection (ICSI) patients. Oocytes were considered mature if they reached meiosis II (MII) stage within 4 hours after oocyte retrieval. The total number of embryos was calculated by counting the embryos after visualization of the 2 pronuclei. Embryos with at least 5 blastomeres and less than 20% of fragmentation on day 3 were classified as good quality embryos [17]. A maximum of two embryos were transferred into the uterus guided by abdominal ultrasound visualization on days 3 or 5, according to the number and evolution of the embryos. Six hundred micrograms of micronized progesterone were used for luteal support. A positive βhCG test (≥10 IU/L) was conducted 12 days after embryo transfer.

With regard to the controlled ovarian stimulation response, we considered as Poor response < 3 growing follicles or less than 4 collected oocytes according to the ESHRE consensus (2011) on the definition of poor responders by Ferraretti et al. [18], Satisfactory response between 4 to 12 follicles, Hyperresponse between 13 to 19 follicles, and Ovarian Hyperstimulation Syndrome (OHHS) with 20 or more follicles or/and clinical symptoms such as ascites, hematological changes (hemoconcentration, hypercoagulation), pleural effusion, and liver abnormalities according to the classifications proposed by Golan et al. [19].

### Analysis of FSH, Estradiol and AMH

Serum FSH, Estradiol (E) and AMH levels were measured on the initial day of the rFSH/COH. FSH and E were mensured by radioimmunoassay (RIA) using commercial RIA kits (ELFA, Enzyme Linked Fluorescent Assay, Mini-VidasBioMerieux, Hazelwood, Missouri, USA). AMH was measured by enzyme-linked immunosorbent assay (ELISA) using the AMH Gen II kit by Beckman Coulter, Inc (Brea, CA, USA). The measurement was realized by automated Labotech (Alka Technology®). Values are presented in concentration of ng/ml (convert to SI units: 1 ng/ml = 7,14pM). The analytic sensibility was 0.008 ng/ml.

### Genotyping

Peripheral blood was collected from each patient in an EDTA-containing tube. Genomic DNA was extracted from lymphocytes of peripheral blood according to the salting out standard protocol (Lahiri and Numberger, 1991). Detection of the polymorphisms *Pvull* and *Xbal* of the *ESR1* gene, and *AluI* and *RsaI* of the *ESR2*gene was performed using the TaqMan system by real-time polymerase chain reaction (PCR). Primers and probes were commercially available and provided by Life Technologies (Foster City, California, USA). Assays were performed with TaqMan Universal Master Mix with 50 to 100 ng of DNA per reaction. PCR conditions were 40 cycles of denaturation at 95°C (15 seconds) and anneal/extension at 60°C (1 minute). A random subset (*20% of samples) was repeatedly genotyped in order to avoid false results, and all the samples had concordant findings.

### Statistical analysis

The analyses were performed using SPSS® 18.0, considering a p value lower than 0.05 or 5% as statistically significant. Allele and genotypic frequencies were calculated by allele counting, and the Hardy-Weinberg (H-W) Equilibrium for each polymorphism was tested using the Chi-square test.

The *Shapiro-Wilk* test was used to assess the distribution of the numerical variables. Than for the association of the numerical variables with the genotypes we used Kruskal-Wallis/Mann–Whitney tests for the no parametric variables and one way ANOVA/Student's t tests for the single parametric variable – FSH. To calculate the level of significance, we used the average for the parametric variables and the median for the no parametric variable.

We investigated the associations between all tested SNPs and overall ovarian stimulation outcomes under the different genetic models. Genotypic Model (AA versus Aa versus aa), Dominant Model (AA + Aa versus aa), and Recessive Model (AA versus Aa + aa). The Bonferroni correction was applied for the Genotypic Model and we now considered p value lower than 0,016 (0,05/3) as statistically significant.

Chi-square or Fisher's Exact tests were used to associate the genotype frequencies of the polymorphisms and the categorical variables regarding ovarian stimulation outcomes.

Furthermore, linkage disequilibrium (LD) among the studied polymorphisms located in the *ESR1* gene (rs2234693 and rs9340799) and the*ERS2* gene (rs4986938 and rs1256049) was measured by D’ using Haploview software. The Expectation-Maximization algorithm was calculated to estimate haplotype frequencies; haplotypes with a frequency less than 0.01 were excluded from the analysis. PLINK software was used to associate haplotypes with both numerical and categorical variables of the study.

## Results

### Characteristics of patients and COH outcome

A total of 136 women were included in this study once they meet the inclusion criteria. The mean age of the patients was 32 ± 3.5 years. The mean baseline FSH and estradiol levels on D3 of the cycle were 6.2 ± 1.7mIU/mL and 50.6 ± 33.8 pg/mL, respectively. The mean number of follicles and oocytes was, respectively, 6.7 ± 4.8 and 6.5 ± 4.7 per patient. The mean number of embryos was 4.2 ± 3.2, with 2.7 ± 2.5 embryos classified as good quality embryos.

According to the COH protocol, 104 patients used rFSH at 100 IU per day and were classified as the homogenous protocol group. Considering the total group, patients used an average of 9.2 days of medication and 1144 IU of rFSH per cycle.

Regarding the classification of the COH response, 63.2% presented with a satisfactory response, 6.6% showed a hyperresponse, and 4.4% developed the OHSS, while 25.7% were classified as poor responders. The fertilization rate was 67% and the pregnancy rate was 32.3% per embryo transfer.

The characteristics of patients and COH outcome did not differ significantly according to the infertility factor.

### Polymorphism analysis

The genotype distribution and allelic frequency of the four single nucleotide polymorphisms are summarized on Table 1. Genotype frequencies observed during this study were in Hardy-Weinberg equilibrium.

**Table 1:** **Incidence of the polymorphisms** ***PvuII*** **,** ***XbaI*** **,** ***RsaI*** **, and** ***AluI*** **genotypes and alleles in the studied population**

**Polymorphism**	**Genotype**	**N**	**%**
*PvuII* C/T - rs2234693	TT	26	19.1
	TC	67	49.3
	CC	43	31.6
	T	119	44
	C	153	56
*XbaI* A/G - rs9340799	AA	57	41.9
	AG	60	44.1
	GG	19	14
	A	174	64
	G	98	36
*Alu* G/A - rs4986938	GG	49	36
	GA	71	52.2
	AA	16	11.8
	G	169	62
	A	103	38
*RsaI* G/A - rs1256049	GG	121	89
	GA	14	10.3
	AA	1	0.7
	G	256	94
	A	16	6

There was a significant association between *PvuII* with days and dose of medication. Individuals with the TT genotype used a bigger amount of rFSH (IU of rFSH) than of TC and CC, both when genotypes were compared alone: TT 1309 IU versus TC 1107 IU (p = 0.006) and versus CC 1105 IU (p = 0.008), and when the recessive model was used: TT 1309 IU versus TC + CC 1106 IU (p = 0.03). After confirming these results in the analyses of the homogenous dose group with the 100 IU/day protocol, we also found that the TT genotype used more days of medication than TC and CC (p = 0.009) (Table 2) and TC + CC (p = 0.005) (Table 3).

**Table 2:** **Association of the** ***PvuII*** **genotypes separately and the outcome of COH/IVF in the homogenous rFSH dose group (100 IU per day)**

**Variables**	***Pvu*** **II C/T - rs2234693**													
	**TT**						**TC CC**					***χ*** ^**2**^ **p**		
	**N**	**Median**	**Minimum**	**Maximum**	**N**	**Median**	**Minimum**	**Maximum**	**N**	**Median**	**Minimum**	**Maximum**		
Age (years)	16	33,5	27	37	54	32	26	37	35	31	24	37	0,41	0,521
BMI (kg/m2)	14	22,23	16,8	28,73	52	24,46	17,71	31,62	35	23,9	19,49	31,14	2,72	0,26
Estradiol (pg/ml)	16	39,67	27,06	253,58	54	46,445	28,14	346,9	35	45,53	28,66	90,22	1,99	0,37
Follicles	16	4	0	15	54	6	0	19	35	5	0	28	0,97	0,62
Oocytes	15	4	0	15	49	6	0	19	33	5	0	28	1,13	0,57
Mature oocytes	15	4	0	15	49	4	0	17	33	4	0	25	0,89	0,64
Embryos	13	4	0	14	43	3	0	14	30	4	1	17	1,66	0,44
Good quality embryos	12	1,5	0	7	43	2	0	11	30	3	0	12	1,23	0,54
Days of rFSH	13	10	9	14	45	9	7	13	31	9	8	12	8,06	0,018*
Dose of rFSH (UI)	13	1000	900	1400	45	900	700	1300	31	900	800	1200	8,06	0,018*

N: Number of Individuals; SD: Standard deviation; *p < 0.05. Results of Kruskal-Wallis (represented by *χ* ^2^).

**Table 3:** **Association of the** ***PvuII*** **genotypes and the outcome of COH/IVF in the homogenous rFSH dose group (100 IU per day) in the recessive model**

**Variáveis**	***Pvu*** **II C/T - rs2234693**									
	**TT**						**TC + CC U p**			
	**N**	**Median**	**Mínimum**	**Maximum**	**N**	**Median**	**Minimum**	**Maximum**		
Age (years)	16	33,5	27	37	89	32	24	37	466	0,16
BMI (kg/m2)	14	22,23	16,8	28,73	87	24,24	17,71	31,62	466	0,16
Estradiol (pg/ml)	16	39,67	27,06	253,58	89	45,83	28,14	346,9	555	0,16
Follicles	16	4	0	15	89	6	0	28	619,5	0,41
Oocytes	15	4	0	15	82	5	0	28	512	0,30
Mature oocytes	15	4	0	15	82	4	0	25	522	0,35
Embryos	13	4	0	14	73	3	0	17	440,5	0,68
Good quality embryos	12	1,5	0	7	73	2	0	12	422,5	0,84
Days of rFSH	13	10	9	14	76	9	7	13	259,5	0,005*
Dose of rFSH (UI)	13	1000	900	1400	76	900	700	1300	259,5	0,005*

N: Number of Individuals; SD: Standard deviation; *p < 0.05. Mann–Whitney results represented by U.

Regarding the XbaI polymorphism we found statistical significant association in the AA genotype and better results of IVF, both in the total group and in the homogeneous protocol group (protocol of rFSH 100UI/day). This results include a larger number of follicles, mature oocytes, embryos and good quality embryos. In the total group, both the recessive and the dominant model reached statistical significance, but this results were confirmed in the homogeneous group only in the recessive model (Table 4). Interestingly, we also observed, in the total group, that the GG genotype used a bigger dose of rFSH in the dominant model: GG 1200UI ± 430.1 versus AG + AA 1134.7UI ± 398.4 (p = 0.035).

**Table 4:** **Association of the** ***XbaI*** **genotypes and the outcome of COH/IVF in the homogenous rFSH dose group (100 IU per day) in the Dominant Model**

**Variáveis**	***XbaI*** **A/G -** ***rs9340799***									
	**AA**						**AG + GG U p**			
	**N**	**Median**	**Mínimum**	**Maximum**	**N**	**Median**	**Minimum**	**Maximum**		
Age (years)	41	32	24	37	64	34,5	28	38	439	0,061
BMI (kg/m2)	41	23,81	17,71	31,14	60	24,46	16,8	31,62	1077,5	0,29
Estradiol (pg/ml)	41	43,98	27,06	88,91	64	46,035	28,14	346,9	1224	0,56
Follicles	41	6	0	28	64	5	0	18	1009	0,046*
Oocytes	38	5,5	0	28	59	5	0	18	859,5	0,052
Mature oocytes	38	5	0	25	59	4	0	17	840,5	0,037*
Embryos	35	4	2	17	51	3	0	14	558,5	0,003*
Good quality embryos	35	3	0	12	50	1	0	11	562	0,004*
Days of rFSH	36	9	8	13	53	9	7	14	813,5	0,22
Dose of rFSH (UI)	36	900	800	1300	53	900	700	1400	813,5	*0,22*

N: Number of Individuals; SD: Standard deviation; *p < 0.05. Mann–Whitney results represented by U.

Relative to the RsaI, there was a significant association between the genotypes and days of medication both in the total (p = 0.001) and the homogenous group (p = 0.011), demonstrating that the wild GG genotype used more days of medication. Besides, GG patients of the homogenous group used also a bigger amount of rFSH (p = 0,005) and although there was just one women with the AA genotype, the results remain the same with the recessive model: GG versus AG + AA (Table 5).

**Table 5:** **Association of the** ***RsaI*** **genotypes and the outcome of COH/IVF in the homogenous rFSH dose group (100 IU per day) in the Recessive model**

**Variáveis**	***RsaI*** **G/A- rs256049**									
	**GG**						**GA + AA U p**			
	**N**	**Median**	**Mínimum**	**Maximum**	**N**	**Median**	**Minimum**	**Maximum**		
Age (years)	95	32	24	37	10	35	31	37	275,5	0,029**
BMI (kg/m2)	92	24,115	17,71	31,62	9	24,34	16,8	28,71	355,5	0,49
Estradiol (pg/ml)	95	42,11	27,06	346,9	10	49,395	40,3	253,58	292,5	0,046*
Follicles	95	5	0	28	10	5	0	13	423	0,57
Oocytes	89	5	0	28	8	6,5	0	13	338	0,81
Mature oocytes	89	4	0	25	8	4	0	11	343,5	0,87
Embryos	79	3	0	17	7	3	1	5	247	0,64
Good quality embryos	78	2	0	12	7	1	0	3	190,5	0,18
Days of rFSH	82	9	7	14	7	8	7	9	107,5	0,005*
Dose of rFSH (UI)	82	900	700	1400	7	800	700	900	107,5	0,005*

N: Number of Individuals; SD: Standard deviation; *p < 0.05. Mann–Whitney results represented by U.

** Individuals with the allele A had better results of COH even thought their average age values were higher than individuals with the genotype GG.

Finally, the analyses of the AluI polymorphism showed that in the total group, the GG genotype had a statistically significant increased frequency of OHSS: 83.3% (p = 0,03) when compared with GA: 16.7% and AA: 0% (p = 0.03).

There were no differences between patients classified as poor responders and normal responders with respect to genotype distribution.

The haplotype analysis of the *ERS1* (PvuII/XbaI) showed that patients with the CA haplotype increased by 0.68 the number of good quality embryos compared with individuals without this combination. Additionally, the TG haplotype decreased by 0.71 the number of good quality embryos (p = 0.04). Since we included male factor infertility, the results including number and quality of embryos should be reviewed in future assessments.

According to the variable age there was no significant difference between the age of the patients and the different genotypes of the polymorphisms that could compromise the COH results.

## Discussion

The prior results, regardless of ER gene polymorphism and their relation with the IFV outcome, are still controversial. This might be due to the heterogeneity of the studied populations regarding the ovarian reserve and the varied exposures including different doses of recombinant FSH for controlled ovarian hyperstimulation.

Ovarian stimulation can be viewed as a dynamic test for the resting ovarian follicle pool [20]. Secondary to the decline in follicle pool with age, the ovarian response to FSH diminishes with age [21]. The European Society of Human Reproduction and Embryology (ESHRE) consensus of poor response, published in 2011, compared 3,825 women entering the first cycle of IVF, showing that the cancelation rate and the occurrence of poor responders (<4 oocytes) increases with age. They identified more than 50% of poor response in women over 40 years of age [18].

It is clear, therefore, that when patients 40 years old or more are included in a group with younger women in order to compare ovarian response with genetic markers, the results will be compromised because of the difference in the response with increased age.

As we know that follicle depletion increases six times after 39 years of age [3,22], and most clinical trials considered important changes in ovarian response after 38 years of age [23,24], we excluded patients more than 38 years of age in the present study.

Also, a recent published study revealed that when patients classified as poor responders with a COH dose of 100 IU of rFSH started a new cycle with 200 IU of rFSH, only 10% continued to be classified as poor responders, while 85% became regular responders (≥3 ≤ 19 follicles), with 5% even developing an OHSS [25]. This shows that patient response can totally change if we increase the dose of rFSH. This finding confirms the huge importance of using fixed dose protocols when you want to compare the ovarian response for COH. In our study we first included patients with 100 and 200 IU of rFSH because the definitions of poor response in literature do not consider the same dose of medication used for controlled ovarian hyperstimulation, and even in the 2011 ESHRE consensus of poor ovarian response, only protocols with at least 150 IU of rFSH per day were available for comparison. However, in our view, the ovarian response for COH should be compared according to the dose of rFSH used for induction, as it can totally change the results, as discussed earlier. In order to avoid this bias, we chose to separate a homogenous group with a fixed dose of 100 IU of rFSH per day and to analyze for the first time the relation between the ER polymorphisms and this COH protocol. Unfortunately, in order to focus on an optimal selection of the study sample, we faced one of the obstacles present in most pharmacogenetic studies, i.e., a small sample size.

Nevertheless, we believe that the heterogeneity in the age of the patients and the dose of rFSH for COH might be responsible for the wide range of results.

The first pharmacogenetic investigation in COH/IVF, by Georgiou et al. in 1997, was focused on the *ESR1* gene polymorphisms [26]. They studied 100 women of 25 to 35 years of age with tubal or unexplained infertility and 100 controls. All patients used the same protocol of COH with 225 IU of gonadotrophins per day. They found that the PvuII TT genotype was associated with decreased pregnancy rates when 2–3 consecutives cycles were analyzed. Since then, there have been few studies comparing the relation between ER polymorphisms and the COH/IVF outcome.

In 1999, Sundarranjan et al. found an association between PvuII CC and a smaller number of follicles, oocytes, embryos, and pregnancy rate. The studied group consisted of unexplained infertility patients between 24 and 39 years of age using a 200 IU rFSH daily COH protocol, but increasing the dose to 300 IU when the follicular number was not adequate [27].

Controversially, Altmae et al., in 2007, found better results of IVF in the PvuI CC genotype in relation to the number of follicles, oocytes, and the level of estradiol on the day of hCG. They also described an association between the C allele and smaller doses of rFSH per good quality embryo. The authors showed that patients with XbaI GG had higher estradiol levels on the day of hCG and estradiol levels per follicle, than the AA genotype. However they included patients up to 45 years of age, a fact that can change the results of COH as discussed earlier. Their group also included patients with endometriosis [28]. It has been suggested that endometriosis might be a leading cause of ovarian dysfunction due to alterations in estrogen and progesterone gene expression [29]. Therefore, in our view, patients with endometriosis should be excluded when comparing the results of COH/IVF, since there is a discussion as to impaired ovarian reserve due to the disease, especially in moderate and severe degrees.

Sequentially, in 2009, Ayvaz et al. confirmed PvuI CC as a prognostic marker for better results of IVF, including the number of mature oocytes, pregnancy rate, and embryo quality. The authors compared 107 fertile women with 104 unexplained infertility patients and described an association between infertility with the genotypes TT and TC for the PvuI and AA XbaI polymorphisms [30].

A prior study by De Castro et al., in 2004, presented an original multilocus analysis of *FSHR* 680 – *ESR1* PvuII - *ESR2* AluI. Similar to our group, they analyzed 170 patients 33 ± 2.55 years of age with a dose of 100–200 IU rFSH using a protocol for COH including tubal and male factor infertility. The comparison was made between poor responders (<4 follicles) and high responders, defined by the author as patients with a follicle production higher than the mean value of ovarian follicles in the total cohort (>10 follicles). The *FSHR* Ser – PvuII T – AluI G was better represented in poor responders [31]. Unfortunately, XbaI was not included in their study.

Anagnoston et al. had totally different results in 2012 in their multigenetic model with *FSHR* Asn/Asn – *ERS1* PvuII CC presenting the worst profile of ovarian induction in 109 IVF women [32]. Furthermore, a different approach was used by Anagnoston in 2013 when he evaluated the number of mutated alleles of PvuI and XbaI and the results of COH/IVF in 203 women. The mutated alleles C (Pvul) and G (Xbal) were associated with the worst quality embryos and smaller estradiol levels on the hCG day [33]. To prepare this analysis they considered that the two genes had necessarily the same behavior as wild and mutated alleles, a fact that has not been confirmed. They also included women up to 45 years of age and endometriosis.

Boudjenah et al. [9] published a study with thirteen SNPs that reportedly influence the outcome of IVF and 427 women. They presented a positive association between the AluI GG and the number of oocytes and estradiol levels on the hCG day. They also selected a homogenous group of 112 women under 38 years of age without endometriosis and found that patients with AluI AA required more exogenous gonadotrophins for COH.

In the present study we demonstrated an association between the amount of medication used for COH and the PvuII polymorphism where patients with PvuII TT used a larger amount of rFSH. Even when we separated a group with exactly the same dose of rFSH per day (100 IU daily), we found more days of use and consequently a larger total dose of rFSH in the TT genotype. These results are in agreement with prior findings by Georgiou et al. [26], Altmae et al. [27], and Ayvaz et al. [30], which showed the PvuII CC genotype improving response and follicular quality, as had been described before [26,28,30], resembling De Castro et al.[31] who described the Pvull C allele frequency as being lower among poor responders (≤ 3 follicles).

Our study is the first to find an association between the XbaI and the COH outcome. The AA genotype is strongly associated with better results showing more follicles, oocytes, embryos, and good quality embryos. This association was seen even in the total group as in the group with the homogeneous dose. Moreover, in the total group we observed a larger amount of rFSH used for COH in the GG genotype.

Compared with the three studies that also analyzed the *ERS2* XbaI, Anagnostou et al. [33] similarly showed association between the mutated G allele with worst quality embryos and lower levels of estradiol on the hCG day, which is different from Altmae et al. [28], who found a larger dose of estradiol on the hCG day and per follicle in the GG genotype [28,33]. As discussed earlier, the difference in the results might be due to the exclusion of patients with endometriosis and older than 38 years of age by our study, as it can significantly change the response to COH. Furthermore, we conducted the first study with a fixed rFSH dose protocol of 100UI per day.

Ayaz et al. [30] found a better fertilization rate in the GG genotype and association between XbaI AA and infertility. As we included male infertility our fertilization rate was not considered, but the frequency of the genotypes of our patients was in accordance with the infertility group of their study.

Difference in ethnicity of the population group could also play a role in contributing to the variation. Screening of the *ESR2* RsaI was seen in the Altmae et al. [28] study in an Estonian population, and the frequency of the genotypes is in agreement with our study, with a rare occurrence of the mutated homozygous AA (0.7%). We had only one patient with this genotype, which can interfere with the statistical results. Nonetheless, we found a statistical association between RsaI GG and a larger amount of rFSH used for ovarian induction. No other associations were found in literature.

As to AluI polymorphism, the frequency of the genotypes could be compared with the Spanish group of De Castro et al. [31]. Although their study found no association when analyzing the polymorphism alone, they described an association with poor responders in the multilocus model FSHR Ser – PvuII T – AluI G [31]. Controversially, we had a statistical relation between AluI GG and HOSS. Our results were similar to Boudjenah et al. [9] who found an association between AluI GG and better results of COH and IVF in comparasion to AluI AA.

The *FSHR* gene is the genetic factor studied most often regarding COH. It was observed that the *FSHR* 680Ser variant is associated with elevated baseline FSH levels and elevated gonadotropin requirements during COH [1,31,34]. Estrogens extend the action of FSH on granulose cells by promoting their proliferation and increasing their expression of FSH receptors [11]. Thus, a multilocus rather than a marker-by-marker statistical analysis might be a promising predictive tool for COH outcome, but the small size of the samples are a limitation factor for this kind of analysis. Although it is very difficult to select a sample of women with the same characteristics regardless of the infertility factor and ovarian reserve, using the exact same dose of medication for COH, differences in these characteristics can totally change the results.

## Conclusion

*ER* gene polymorphisms are associated with the results of COH. *ERS1* XbaI AA is associated with better results of COH, and AluI GG is associated with OHSS, while PvuII TT, XbaI GG, and RsaI GG are associated with a larger amount of rFSH in IVF cycles. More studies with larger samples are needed to confirm these results. Also, clinically well selected samples taking into account different COH protocols with the same dose of medication would be necessary in order to compare the COH outcomes, increasing the possibility of a true association.
